# RIN1 regulates developmental and pain-related plasticity in spinal synapses via NMDA receptor subunit trafficking

**DOI:** 10.1371/journal.pbio.3003516

**Published:** 2025-12-02

**Authors:** Hu-Hu Bai, Qi Zhang, Shu-Jin Wu, Yu-Bo Gao, Juan Li, Xue Bai, Xu Yang, Xiao-Xue Liu, Jia-Ning Dang, Xian Yang, Zhan-Wei Suo, Xiao-Dong Hu

**Affiliations:** 1 Department of Molecular Pharmacology, School of Pharmacy, Lanzhou University, Lanzhou, Gansu, P.R. China; 2 Department of Pharmacy, Gansu Provincial Hospital, Lanzhou, Gansu, P.R. China; Columbia University Irving Medical Center, UNITED STATES OF AMERICA

## Abstract

Neuronal activity and sensory experience regulate the subunit stoichiometry of synaptic N-methyl-D-aspartate subtype glutamate receptors (NMDARs), a critical determinant for brain development, synaptic plasticity, and a line of neurological disorders. Here we found that Ras and Rab interactor 1 (RIN1), a neuron-specific protein in the brain, played an important role in dictating synaptic NMDAR subunit composition in spinal cord somatostatin-positive (SOM^+^) neuron, a key component in the spinal circuit transmitting mechanical pain in mice. Our data showed that the protein level of RIN1 was low early after birth, which progressively increased with synapse maturation and promoted the switch from synaptic GluN2B- to GluN2A-containing NMDARs. In adult mice, the nerve injury-induced pathological pain paralleled a significant increase of RIN1 protein in spinal SOM^+^ neurons, which drove a new round of GluN2B-to-GluN2A switching at mature synapses. Our data revealed the molecular mechanisms by which RIN1 differentially regulated the synaptic trafficking of GluN2B and GluN2A receptors, and implied that RIN1-mediated pathological switch of NMDAR subunit composition strikingly altered the analgesic efficacy of distinct NMDAR subunit antagonists with the development of neuropathic pain.

## Abstract 2

Modifications in the subunit composition of NMDA receptors are important for regulating synaptic function, but how are these changes controlled? This study in mice shows that the protein RIN1 differentially regulates the localization of GluN2B and GluN2A receptor subunits at spinal synapses, providing insights into synaptic changes during development and neuropathic pain.

## Introduction

N-Methyl-D-aspartate subtype glutamate receptors (NMDARs) in spinal cord dorsal horn are critically involved in pain modification [1]. The majority of NMDARs are made of obligatory GluN1 and regulatory GluN2 (GluN2A to GluN2D) subunits [2]. Abundant evidence has revealed an enhanced nociceptive transmission mediated by GluN2B subunit-containing NMDARs (short for GluN2B receptors) after nerve or tissue injury, and demonstrated a potent analgesic action of GluN2B-selective antagonists [3–7]. The NMDARs containing other GluN2 isoforms, including GluN2A or GluN2D subunits, have also been detected in spinal cord [8–10]. The specific NMDAR subunit composition at a given spinal synapse remains to be elucidated.

The synaptic contribution of individual NMDAR isoforms varies with the afferent inputs, neuron subtypes, pathophysiological contexts, and developmental stages [3,11,12]. For example, the primary afferent-evoked monosynaptic NMDAR currents on lamina I projection neurons are predominantly mediated by GluN2A subunit-containing NMDARs, whereas the polysynaptic currents are largely mediated by GluN2B receptors [11]. Pathological pain correlates with a substantial accumulation of GluN2B receptors at primary afferent synapses, leading to the amplification of nociceptive signals [3,4]. The lamina I neurons also receive the inputs from local glutamatergic interneurons, with some synapses expressing GluN2B and GluN2D subunits [13]. The developmental modification of NMDAR subunit composition is well-characterized in the brain. The GluN2B receptors are prevalent neonatally [1,12,14,15]. With synapse development, GluN2B receptors are gradually replaced by GluN2A receptors [1,16–21]. This developmental switch speeds up the decay kinetics of NMDAR currents and modulates the thresholds for synaptic plasticity [12,19,22–24]. However, whether the primary afferent synapses onto genetically defined spinal nociceptive transmission neurons exhibit the canonical developmental switch remains elusive [4,25].

The spinal dorsal horn consists of excitatory and inhibitory interneurons as well as a small number of molecularly and functionally distinct projection neurons engaging different brain regions for sensory and affective processing [26–28]. The synaptic conveyance of mechanical allodynia, the most prevalent and debilitating disorder in patients suffering from nerve injury, largely depends on a subpopulation of mechanosensory interneurons positive for somatostatin (SOM) [29–32]. Genetic ablation of SOM^+^ neurons completely abolishes mechanical allodynia in pathological pain conditions [30,31], whereas other interneurons, such as those expressing calretinin, preprotachykinin 2 and neuropeptide Y, are dispensable for mechanical pain [30]. Spinal SOM^+^ interneurons receive feedforward inhibition from local GABAergic interneurons, whose dysfunction also contributes to mechanical allodynia [33,34].

Given the central role of SOM^+^ interneurons in the relay of mechanical signals from the periphery to ascending pain transmission pathway, the current study investigated the NMDAR subunit composition at primary afferent synapses onto SOM^+^ interneurons, and identified Ras and Rab interactor 1 (RIN1), an intracellular signaling protein implicated in the negative control over synaptic plasticity [35,36], as an important determinant of synaptic NMDAR subunit stoichiometry. RIN1 can interact through its N-terminal Src homology-2 (SH2) domain with a line of receptor tyrosine kinases. The vacuolar protein sorting 9 protein (Vps9p) domain, located at the C-terminal region of RIN1, exhibits the guanine nucleotide exchange factor (GEF) activity for small GTPase Rab5 critical for protein endocytosis [37–39]. We found that RIN1 expressed in SOM^+^ interneurons played an important role in the determination of synaptic NMDAR subunit composition during both the development and neuropathic pain.

## Results

### RIN1 was involved in the developmental switch of synaptic NMDA receptors

**Fig 1:**
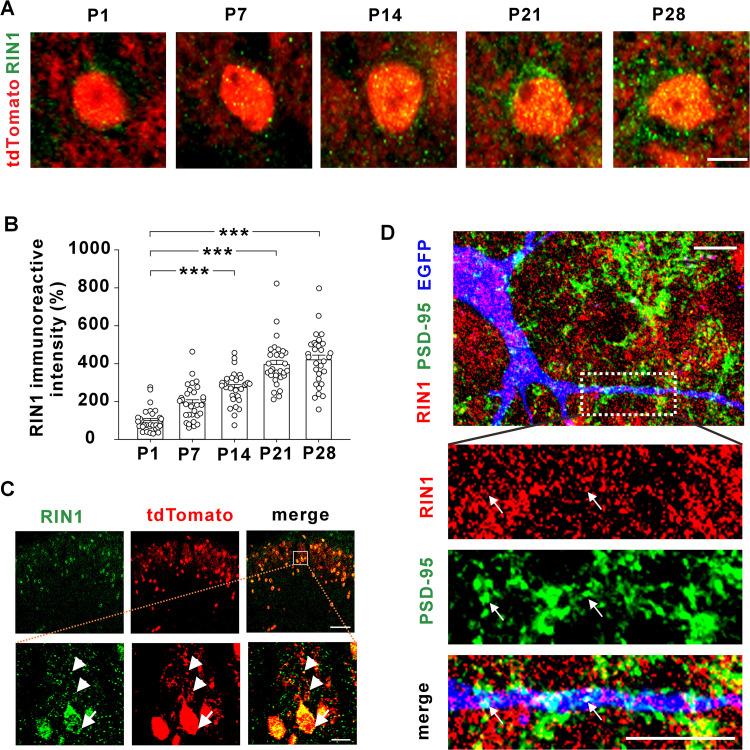
Expression of RIN1 in spinal cord SOM^+^ interneurons. **(A and B)** Immunohistochemistry for RIN1 (green) in spinal sections of SOM-tdTomato mice at postnatal day 1 (P1) to P28. ***P < 0.001 (Kruskal–Wallis test). *n* = 30 neurons from 3 mice per group. Scale bar, 5 µm. **(C)** Distribution of RIN1 (green) in the soma (arrow) and dendrites (arrowhead) of tdTomato^+^ neurons in the superficial dorsal horn of adult SOM-tdTomato mice. The area enclosed in the white square (*up*) was enlarged (*down*). Scale bar, 100 µm (*up*) and 10 µm (*down*). **(D)** Immunohistochemistry for RIN1 (red) and PSD-95 (green) in SOM^+^ neurons (blue) labelled by intraspinal injection of AAV2/9-DIO-EGFP in SOM-Cre mice. Arrows indicated the colocalization. Scale bar, 10 µm. The data underlying this Figure can be found in S1 Data.

RIN1 protein is abundant in several forebrain structures of adult mice [35,36]. To examine the distribution of RIN1 in spinal cord somatostatin-positive (SOM^+^) interneurons, we crossed SOM-Cre mice with Ai14 reporter mice to label SOM^+^ neurons with tdTomato (hereafter SOM-tdTomato mice). Consistent with previous studies on forebrain regions showing a developmental expression of RIN1 [35,36], the protein levels of RIN1 in spinal SOM^+^ interneurons increased from postnatal day 1 (P1), which reached a plateau at P21 and remained constant at P28 (Fig 1A and 1B). Dense punctate RIN1 signals were observed in the soma and dendrites of SOM^+^ neurons in the superficial dorsal horn of adult mice (Fig 1C). To test whether RIN1 was present at excitatory glutamatergic synapses, we labeled SOM^+^ neurons by intraspinal injection of a Cre-inducible adeno-associated virus (AAV) carrying enhanced green fluorescent protein (EGFP) in SOM-Cre mice (Fig 1D). Double immunofluorescence showed that 44% of RIN1 signals were coincident with PSD-95 (Fig 1D), a postsynaptic density marker, and 75% of PSD-95 signals were co-localized with RIN1 (*n* = 5 sections from 2 mice).

**Fig 2:**
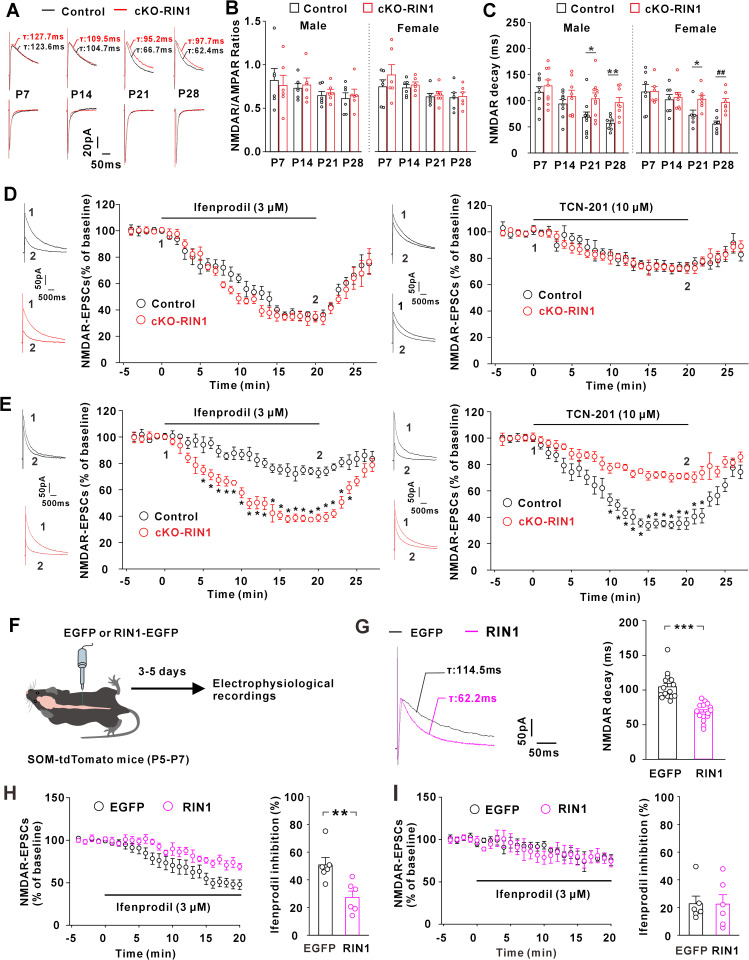
RIN1 contributed to the developmental switch of synaptic GluN2B to GluN2A receptors in spinal cord SOM^+^ interneurons. **(A)** Representative NMDAR-EPSCs and AMPAR-EPSCs recorded from P7 to P28. **(B and C)** Comparisons of NMDAR/AMPAR ratios (B) and decay kinetics of NMDAR-EPSCs (C) recorded from male and female mice. *P = 0.035, **P = 0.005, ^*##*^*P* = 0.002 (Mann-Whitney U test). *n* = 6 neurons (B) and 6-10 neurons (C) from 4-5 mice per group. **(D)** Changes of NMDAR-EPSCs recorded in spinal slices prepared from control and cKO-RIN1 mice at P5-P7 after bath perfusion of ifenprodil (*left*; F(31, 310) = 0.997, *P* = 0.475, repeated measurement, *n* = 6 neurons/group from 3-4 mice) or TCN-201 (*right*; F(31, 310) = 0.491, *P *= 0.991, repeated measurement, *n* = 6 neurons/group from 3-4 mice). **(E)** Changes of NMDAR-EPSCs recorded in control and cKO-RIN1 mice at P28-P35 after bath perfusion of ifenprodil (*left*; F(31, 310) = 7.877, *P* < 0.001, repeated measurement) or TCN-201 (*right*; F(31, 310) = 6.514, *P* < 0.001, repeated measurement). *P < 0.05 (post hoc Bonferroni test). *n* = 6 neurons/group from 3-4 mice. **(F)** Scheme for the injection of an adenoviral vector carrying RIN1 and/or EGFP in spinal cord dorsal horn of SOM-tdTomato mice at P5-P7 and electrophysiological recordings after 3-5 days. **(G and H)** Effects of RIN1 expression on the decay kinetics of NMDAR-EPSCs (G) and ifenprodil sensitivity **(H)**. **P = 0.004, ***P < 0.001 (Mann-Whitney U test). *n* = 15 neurons/group (G) and 6 neurons/group (H) from 4-5 mice. **(I)** Effect of ifenprodil on NMDAR-EPSCs recorded on SOM^+^ interneurons expressing RIN1 or EGFP at P28-P35. *n* = 6 neurons from 2–3 mice/group. The data underlying this Figure can be found in S1 Data.

To test the possible role of RIN1 in synaptic modification, we knocked out RIN1 in SOM^+^ neurons by crossing SOM-Cre, Ai14, and floxed RIN1 (RIN1^*fl/fl*^) mice. The conditional RIN1 knockout mice (referred to here as cKO-RIN1 mice) showed an absence of RIN1 in spinal cord SOM^+^ neurons (S1A Fig). We prepared the acute spinal cord slices from cKO-RIN1 mice at P7-P28, and conducted the whole-cell patch clamp recordings on SOM^+^ interneurons. RIN1 ablation didn’t affect the frequencies and amplitudes of miniature excitatory postsynaptic currents (mEPSCs) mediated by AMPA receptors (AMPAR-mEPSCs) in both male and female mice when compared to the age- and sex-matched control SOM-tdTomato mice (S1B and S1C Fig), suggesting that RIN1 was dispensable for the regulation of AMPAR responses in spinal cord slices. We then assayed the effect of RIN1 on synaptic NMDAR currents by recording and calculating the ratios of NMDAR-EPSCs to AMPAR-EPSCs (NMDAR/AMPAR ratios). Compared to the age-matched control mice, RIN1 deletion generated no detectable changes in the NMDAR/AMPAR ratios from P7 to P28 in the male and female mice (Fig 2A and 2B), suggesting that RIN1 didn’t determine the total number of NMDARs at postsynaptic membranes.

The immature synapses in the brain predominantly contain NMDARs with GluN2B subunits (referred to here as GluN2B receptors) [1,12,14,15]. With synapse development, GluN2B receptors are gradually substituted by those with GluN2A subunits (referred to as GluN2A receptors). Because GluN2B receptors have slower decay dynamics than GluN2A receptors, the decay time constants (τ) of NMDAR-EPSCs exhibited a developmental decrease [1,12]. At primary afferent synapses onto spinal cord SOM^+^ interneurons of control male mice, we also observed a gradual speeding of NMDAR-EPSC decay from P7 to P28 (Fig 2C). RIN1 ablation, however, blunted the developmental change of NMDAR-EPSC decay (Fig 2C). At P21 and P28, the decay kinetics was significantly slower in cKO-RIN1 mice than the age- and sex-matched control ones (Fig 2A and 2C). In female mice, the developmental decline of the EPSC decay was also blocked by RIN1 deletion (Fig 2C), indicating no sex difference in the developmental alteration of NMDAR subunit stoichiometry. The following experiments were conducted in mice of both sexes.

To examine whether RIN1 regulated the GluN2B-to-GluN2A switch, we tested the sensitivity of NMDAR-EPSCs to ifenprodil and TCN-201, the GluN2B- and GluN2A-selective antagonist, respectively. We first prepared the spinal slices from mice at P5-P7, during which RIN1 expression was relatively low in spinal SOM^+^ neurons (Fig 1A and 1B). Extracellular perfusion of ifenprodil (3 μM) and TCN-201 (10 μM) inhibited NMDAR-EPSCs to a similar extent in cKO-RIN1 and control mice (Fig 2D), suggesting that the synaptic abundance of GluN2B or GluN2A was comparable between the phenotypes. We then prepared the slices from mice at the age of P28-P35, during which putative GluN2B-to-GluN2A switch has been shown to reduce synaptic GluN2B accumulation at brain synapses [17,22]. Compared to the control mice, the cKO-RIN1 mice exhibited a higher ifenprodil sensitivity (Fig 2E). TCN-201 sensitivity was, however, reduced in cKO-RIN1 mice relative to control ones (Fig 2E). These results suggested that similar to those central synapses, the primary afferent synapses onto spinal cord SOM^+^ interneurons also exhibited a developmental decrease of GluN2B receptors and a simultaneous increase of GluN2A receptors, a process that required the involvement of RIN1.

To directly examine whether RIN1 accelerated the process of GluN2B-to-GluN2A switch, we injected an adenoviral vector carrying RIN1 and EGFP in spinal cord of SOM-tdTomato mice at P5-P7. After 3–5 days of viral injection, we recorded NMDAR-EPSCs on tdTomato^+^/EGFP^+^ neurons (Fig 2F). Viral expression of exogenous RIN1 at the early stage of development speeded up the decay kinetics of synaptic NMDAR currents (Fig 2G) and reduced the ifenprodil sensitivity when compared to EGFP control (Fig 2H). When neurons matured and RIN1 protein levels peaked at P28-P35 (Fig 1A and 1B), exogenous RIN1 expression had no effect on the ifenprodil sensitivity (Fig 2I). These data implied that RIN1 was prerequisite for the developmental switch of synaptic NMDAR subunit composition at the primary afferent synapses on spinal cord SOM^+^ interneurons.

### RIN1 promoted the GluN2B-to-GluN2A switch through its SH2 and Vps9p domain

**Fig 3:**
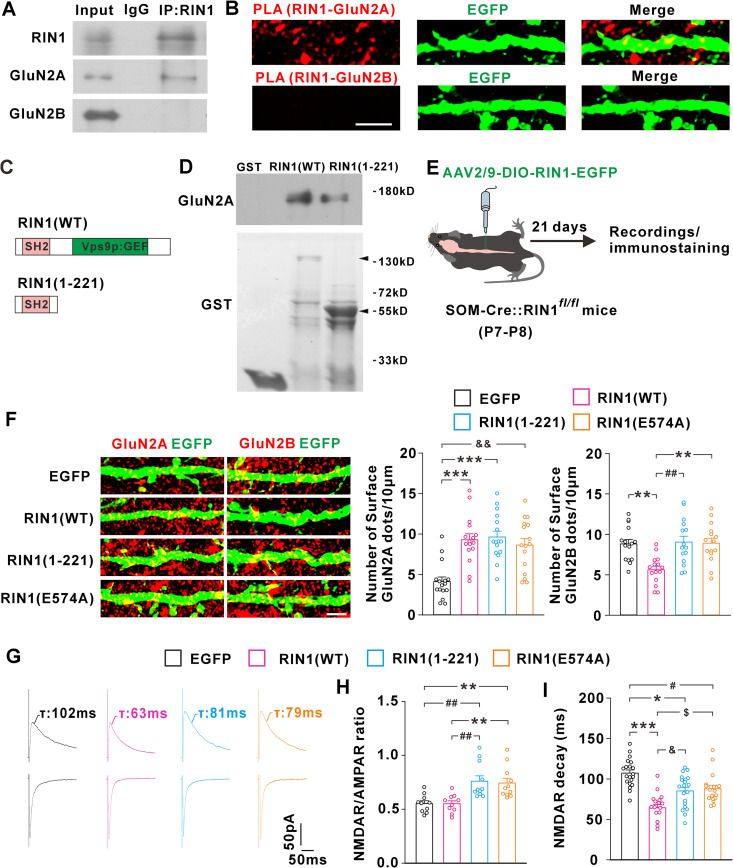
RIN1 promoted synaptic GluN2B-to-GluN2A switch through its SH2 and Vps9p domain. **(A)** RIN1 immunoprecipitates from spinal cord dorsal horn of mice were probed with the antibodies indicated on the left of panels. The experiments were replicated for 3 times and each obtained a similar result. IgG, immunoglobulin G; IP, immunoprecipitation. **(B)** PLA detection of RIN1-GluN2A complex (*up*) or RIN1-GluN2B complex (*down*) in EGFP-labeled SOM^+^ interneurons. Scale bar, 5 µm. **(C and D)** GST fusion of wild-type RIN1 [RIN1(WT)] or RIN1(1-221) mutant (C) pulled down GluN2A from the lysates of spinal cord **(D)**. Arrowheads indicated the apparent molecular size of GST-RIN1(WT) or GST-RIN1(1-221) **(D)**. The experiments were replicated for 3 times and each obtained a similar result. **(E)** Scheme for the viral injection in the spinal cord of SOM-Cre::RIN1^*fl/fl*^ mice at P7-P8 and electrophysiological recordings or immunostaining after 21 days. **(F)** Effects of RIN1(WT) and its mutants on the surface expression of GluN2A and GluN2B. ***P < 0.001, **P = 0.003, ^##^*P* = 0.006, ^*&&*^*P* = 0.002 (Kruskal–Wallis test). *n* = 16 neurons (GluN2A) and 15 neurons (GluN2B) from 5 mice. Scale bar, 4 µm. (G-I) Effects of RIN1(WT) and its mutants on NMDAR/AMPAR ratios (G, H; **P = 0.008, ^*##*^*P* = 0.006; Kruskal–Wallis test; *n* = 10–11 neurons/group from 5-7 mice) and NMDAR-EPSC decay kinetics (G, I; ***P < 0.001, *P = 0.018, ^*#*^*P* = 0.042, ^*&*^*P* = 0.032, ^*$*^*P* = 0.022; Kruskal–Wallis test; n = 18-20 neurons/group from 5-7 mice). The data underlying this Figure can be found in S1 Data.

Protein-protein interaction has been shown to play a crucial role in the dynamic transportation of NMDARs to and from the synapses [40,41]. To explore the mechanism whereby RIN1 regulated synaptic NMDAR composition, we examined the possible interaction between RIN1 and NMDARs. Co-immunoprecipitation showed that RIN1 antibody precipitated GluN2A subunit from the lysates of spinal cord dorsal horn of mice (Fig 3A). The GluN2B subunit was, however, undetectable in the RIN1 immunoprecipitates (Fig 3A). To observe the RIN1 interaction with GluN2A in spinal cord SOM^+^ interneurons, we labeled the neurons by injecting AAV2/9-DIO-EGFP in spinal cord of SOM-Cre mice and conducted the proximity ligation assay (PLA) in the spinal slices. The PLA detection of RIN1-GluN2A proximity produced punctate signals on SOM^+^ neurons (Fig 3B), and these signals were invisible when GluN2B antibody was used (Fig 3B), suggesting that endogenous RIN1 specifically interacted with GluN2A in SOM^+^ neurons. To confirm the direct binding of RIN1 to GluN2A, we performed GST pull-down assays in vitro. GST fusion protein of wild-type RIN1 [GST-RIN1(WT)] stably pulled down GluN2A from the lysates of spinal cord dorsal horn (Fig 3C and 3D). There is a Src homology-2 (SH2) domain at the N-terminal region of RIN1 (Fig 3C), which has been implicated in the binding to several transmembrane proteins [36,42]. Our data showed that GST-RIN1(1–221), a GST-fused N-terminal region encompassing the SH2 domain (Fig 3C), was sufficient to pull down GluN2A (Fig 3D).

To investigate how RIN1 interacts with GluN2A-regulated the GluN2B-to-GluN2A switch, we knocked down RIN1 by crossing SOM-Cre mice with RIN1^*fl/fl*^ mice, and rescued the RIN1 expression in SOM^+^ neurons by targeting a Cre-inducible AAV vector carrying RIN1(WT) and EGFP to spinal cord at P7-P8 (Figs 3E and S2). Three weeks after viral injection, we immunostained GluN2A and GluN2B receptors expressed on the plasma membranes of EGFP-labeled SOM^+^ neurons. Compared to EGFP control, RIN1(WT) increased the surface expression of GluN2A and meanwhile, reduced that of GluN2B (Fig 3F). Electrophysiological recordings showed that RIN1(WT) didn’t affect the NMDAR/AMPAR ratios (Fig 3G and 3H) but significantly speeded up the decay of NMDAR-EPSCs related to EGFP control (Fig 3G and 3I), indicative of NMDAR subunit switch driven by RIN1(WT).

Next, we virally targeted RIN1(1–221) to spinal cord SOM^+^ interneurons. Different from RIN1(WT), the RIN1(1–221) mutant lacking the C-terminal region induced the accumulation of GluN2A on the plasma membrane, which, however, had no effect on the surface expression of GluN2B when compared to the EGFP control (Fig 3F). RIN1(1–221) also enhanced the NMDAR/AMPAR ratios (Fig 3G and 3H), implying an increase of total NMDAR number at synapses. Compared to the EGFP control, RIN1(1–221) speeded up the decay of NMDAR-EPSCs (Fig 3G and 3I), albeit to a lesser degree than RIN1(WT) (Fig 3G and 3I). These data suggested that the N-terminal region of RIN1, through direct binding to GluN2A, subserved the net addition of GluN2A receptors into synapses, which, however, failed to disperse or replace the pre-existing synaptic GluN2B receptors due to the lack of C-terminal tail.

The C-terminal region of RIN1 contains a Vps9p domain, which exhibits GEF activity for Rab5 and regulates the endocytosis of several transmembrane proteins [37,38]. To test the possible role of Vps9p domain in the GluN2B-to-GluN2A switch, we intraspinally injected a Cre-inducible AAV vector coding for RIN1(E574A), a RIN1 mutant in which the glutamic acid at residue 574 was substituted with alanine to abolish Rab5 GEF activity [37,38]. The RIN1(E574A) mutant caused a selective increase of GluN2A surface expression (Fig 3F), coincident with which was the enhanced NMDAR/AMPAR ratios (Fig 3G and 3H) and a moderate acceleration of the decay kinetics of NMDAR currents (Fig 3G and 3I). These data suggested that the GluN2B-to-GluN2A switch might involve two independent but relevant steps: the synaptic incorporation of GluN2A that relied on the N-terminal region of RIN1, and the simultaneous removal of GluN2B from synapses that was attributed to the Rab5 GEF activity.

### Nerve injury induced the switch of NMDAR subunit composition at mature synapses on spinal SOM^+^ interneurons

Mechanical allodynia is the most prevalent and intractable symptom of chronic pain. The spinal transmission of mechanical allodynia is critically dependent on the SOM^+^ subpopulation [29–32]. To confirm the role of SOM^+^ neurons in mechanical pain, we infused a Cre-inducible AAV coding for an inhibitory designer receptor exclusively activated by designer drugs (DREADD) fused to mCherry (hM4Di-mCherry) in spinal cord of SOM-Cre mice before establishing the spared nerve injury (SNI) model of neuropathic pain (S3A Fig). In male mice, the neuropathic mechanical allodynia was completely abolished when the DREADD activator clozapine-N-oxide (CNO; 5 mg/kg) was intraperitoneally injected either at days 7–10 or at days 25–28 post-SNI (S3B Fig). The chemogenetic inhibition of SOM^+^ subpopulation also reversed mechanical allodynia in female mice (S3C Fig), suggesting that the SOM^+^ subpopulation served as the key component in the spinal circuits that relayed the mechanosensory information from the periphery to the ascending pain pathways. Post hoc examination verified that the hM4Di-mCherry was expressed in spinal cord (S3D Fig) and bath perfusion of CNO decreased the excitability of SOM^+^ interneurons (S3E Fig).

**Fig 4:**
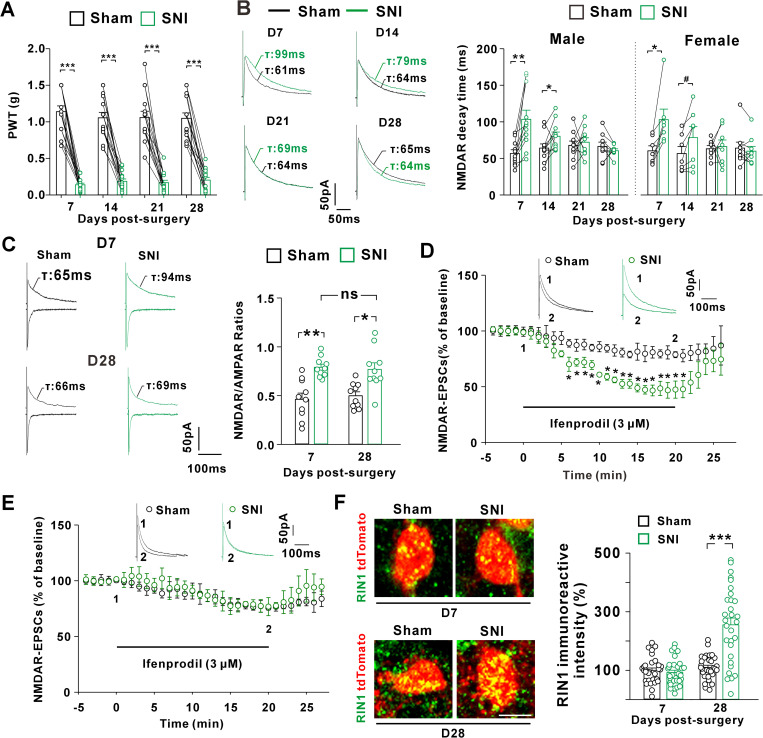
Peripheral nerve injury induced synaptic GluN2B-to-GluN2A switch in spinal cord SOM^+^ neurons of both male and female SOM-tdTomato mice. **(A)** Changes in the paw withdrawal thresholds (PWTs) of male and female mice after spared nerve injury (SNI) on the left sciatic nerves and sham surgery on the right nerves. ***P < 0.001 (paired Student *t t*est). *n* = 15 mice/group. **(B)** Changes in the decay kinetics of NMDAR-EPSC recorded in spinal slices from male mice (*left*; **P = 0.002, *P = 0.03, Paired Student *t t*est, n = 9-11 neurons from 3–5 mice per group) and female mice (*right*; *P = 0.01, ^*#*^*P* = 0.035, Paired Student *t t*est, *n* = 7–9 neurons from 3-4 mice per group). **(C)** NMDAR/AMPAR ratios recorded at days 7 (D7) and 28 (D28) after surgery in male and female mice. *P = 0.028, **P = 0.002, ^*ns*^*P* = 1.00 (Kruskal–Wallis test). *n* = 10 neurons from 3–4 mice per group. **(D)** Inhibition by ifenprodil of NMDAR-EPSCs recorded at day 7 after surgery in male and female mice. F(30, 270) = 1.932, *P* = 0.003 (repeated measurement). *P < 0.05 (Post hoc Bonferroni test). *n* = 6 neurons/group from 3-4 mice. **(E)** Inhibition by ifenprodil of NMDAR-EPSCs recorded at day 28 after surgery in male and female mice. F(31, 310) = 0.421, *P* = 0.998 (repeated measurement). *n* = 6 neurons per group from 3–4 mice. **(F)** Immunohistochemistry for RIN1 (green) in spinal SOM^+^ interneurons at days 7 and 28 after surgery in male and female mice. ***P < 0.001 (Kruskal–Wallis test). *n* = 30 neurons from 3 mice per group. The data underlying this figure can be found in S1 Data.

To test the influence of nerve injury on the NMDAR subunit composition at primary afferent synapses onto the SOM^+^ interneurons, we conducted the SNI and sham surgery on the left and right sciatic nerves of SOM-tdTomato mice, respectively. Both male and female mice exhibited mechanical allodynia at day 7 post-SNI (Fig 4A). We then prepared the spinal slices from the neuropathic mice and recorded NMDAR-EPSCs on the SOM^+^ interneurons. In male and female mice, the decay kinetics of NMDAR-EPSCs was significantly slower on the injured side than sham side (Fig 4B). At day 14 post-SNI, the decay kinetics of NMDAR-EPSCs remained slower on the injured side relative to the sham side (Fig 4B). However, when recorded at the later phase of neuropathic pain (days 21 and 28 post-SNI) (Fig 4A), the decay kinetics became indistinguishable between the SNI and sham sides (Fig 4B). These data suggested that distinct NMDAR subunit composition at primary afferent synapses on spinal SOM^+^ interneurons underpinned mechanical allodynia early and later after the nerve injury, a synaptic modification occurring equally in male and female mice.

Peripheral injury can induce a rapid redistribution of GluN2B receptors at spinal synapses, which enhances NMDAR synaptic currents and leads to NMDAR-dependent pain sensitization [3,4]. Our data demonstrated that the NMDAR/AMPAR ratios (Fig 4C) and the sensitivity of NMDAR-EPSCs to ifenprodil (Fig 4D) were enhanced at day 7 post-SNI, supporting the synaptic accumulation of GluN2B receptors early after nerve injury. The mechanisms that resumed synaptic NMDAR subunit stoichiometry later after nerve injury might involve the addition of more GluN2A receptors into synapses. If this were the case, the amplitudes of NMDAR synaptic currents would be potentiated to a greater degree at the later phase of neuropathic pain. Our data showed that the enhancement of NMDAR/AMPAR ratios was comparable between day 28 and day 7 post-SNI (Fig 4C), arguing against the net addition of GluN2A into synapses. By analyzing the ifenprodil sensitivity at day 28 post-surgery, we found that ifenprodil inhibited NMDAR currents to a similar extent on the sham and SNI sides (Fig 4E), supporting a progressive replacement of synaptic GluN2B receptors by GluN2A receptors with the development of neuropathic pain. Given the importance of RIN1 in driving GluN2B-to-GluN2A switch during synapse maturation, we examined the change of RIN1 expression after nerve injury in SOM-tdTomato mice. Compared to sham control, SNI didn’t affect the RIN1 immunoreactivity on SOM^+^ neurons after 7 days (Fig 4F). However, the RIN1 protein levels were significantly increased at day 28 after SNI relative to sham surgery (Fig 4F), suggesting that enhanced RIN1 expression paralleled with the neuropathic pain-related NMDAR subunit switch at primary afferent synapses onto the SOM^+^ interneurons.

In addition to the excitatory SOM^+^ interneurons, the spinal inhibitory interneurons also receive the synaptic input from primary afferent mechanosensory neurons and drive the feedforward inhibition of SOM^+^ interneurons (S4A Fig). To examine the expression of RIN1 in the inhibitory interneurons, we injected AAV2/9-DIO-EGFP in spinal cord of Vgat-Cre mice before SNI or sham surgery (S4B Fig). Immunohistochemistry showed that the EGFP^+^ inhibitory interneurons indeed expressed RIN1 (S4C Fig). However, the RIN1 contents in either male (S4C Fig) or female mice (S4D Fig) had no significant changes at day 7 or 28 post-SNI when compared to sham control. Recordings of NMDAR-EPSCs on the EGFP^+^ inhibitory interneurons of male (S4E Fig) and female mice (S4F Fig) demonstrated that the decay kinetics were comparable between the SNI and sham sides at either day 7 or 28 after the surgery, indicative of no subunit switch at primary afferent synapses onto the inhibitory interneurons.

**Fig 5:**
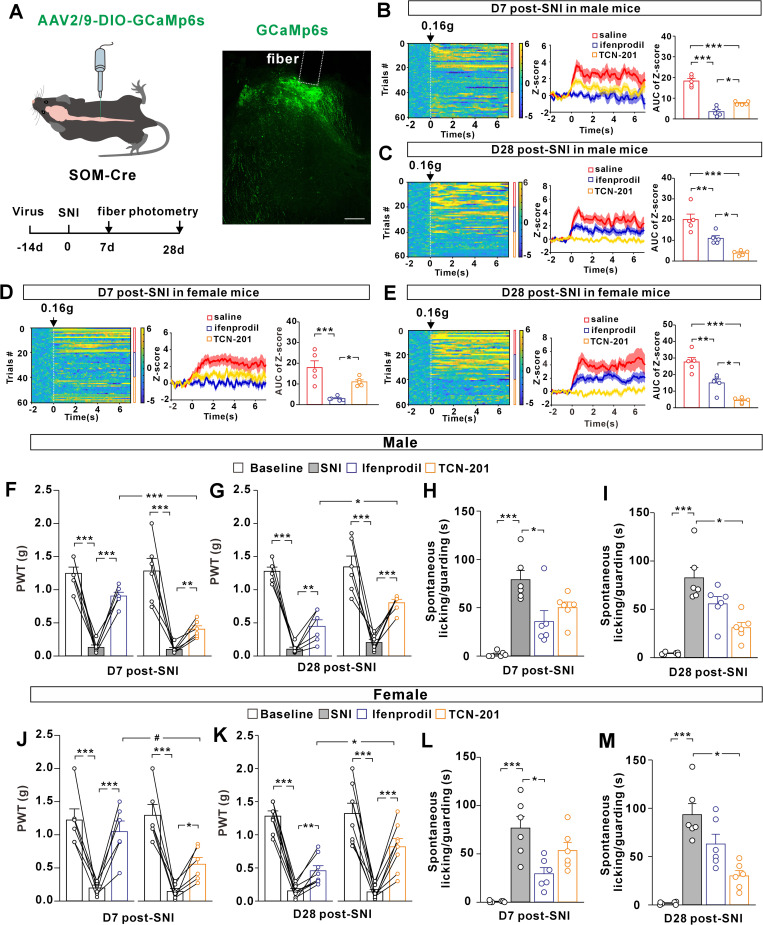
Time-dependent change in the sensitivity of neuropathic pain to GluN2B and GluN2A receptor antagonists. **(A)** Scheme for injection of an AAV vector carrying GCaMp6s in the spinal cord of SOM-Cre mice and experimental schedule (*left*). Fluorescence expression in the spinal cord was shown (*right*). Scale bar, 100 μm. **(B and C)** Effects of intraperitoneal ifenprodil (10 mg/kg) or TCN-201 (10 mg/kg) application on Ca^2+^ responses of spinal SOM^+^ subpopulation to von Frey filament (0.16 g) stimulation of the plantar surfaces of male mice at day 7 (D7, B; ***P < 0.001, *P = 0.028, One-way ANOVA and post hoc Bonferroni test, *n* = 5 mice/group) or day 28 (D28, C; ***P < 0.001, **P = 0.009, *P = 0.039, *n* = 5 mice/group) after spared nerve injury (SNI). Saline was used as control. **(D and E)** Effects of ifenprodil and TCN-201 on Ca^2+^ responses of female mice at day 7 (D7, D; ***P < 0.001, *P = 0.04, One-way ANOVA and post hoc Bonferroni test, *n* = 5 mice/group) or day 28 (D28, E; ***P < 0.001, **P = 0.004, *P = 0.013, n = 5 mice/group) after SNI surgery. **(F and G)** PWTs of male mice after intrathecal application of ifenprodil (10 nmol) or TCN-201 (10 nmol) at day 7 (F; ***P < 0.001, **P = 0.001, repeated measurement with post hoc Bonferroni test, *n* = 6 mice/group) or day 28 (G; ***P < 0.001, **P = 0.006, *P = 0.01, repeated measurement with post hoc Bonferroni test, *n* = 6 mice/group) after SNI. (H and **I)** Spontaneous pain behaviors of male mice after intrathecal ifenprodil or TCN-201 treatment at day 7 (H; ***P < 0.001, *P = 0.027, Kruskal–Wallis test, *n* = 6 mice/group) or day 28 post-SNI (I; ***P < 0.001, *P = 0.013, Kruskal–Wallis test, *n* = 6 mice/group). **(J and K)** PWTs of female mice after intrathecal application of ifenprodil or TCN-201 at day 7 (J; ***P < 0.001, *P = 0.025, ^*#*^*P* = 0.027, repeated measurement with post hoc Bonferroni test, *n* = 6 mice/group) or day 28 (K; ***P < 0.001, **P = 0.009, *P = 0.026, repeated measurement with post hoc Bonferroni test, *n* = 8 mice/group) after SNI. **(L and M)** Spontaneous pain behaviors of female mice after intrathecal ifenprodil or TCN-201 treatment at day 7 (L; ***P < 0.001, *P = 0.03, Kruskal–Wallis test, *n* = 6 mice/group) or day 28 post-SNI (M; ***P < 0.001, *P = 0.013, Kruskal–Wallis test, *n* = 6 mice/group). The data underlying this figure can be found in S1 Data.

Given the critical role of SOM^+^ interneurons in the conveyance of mechanical allodynia, we proposed that the NMDAR subunit recomposition at primary afferent synapses onto the SOM^+^ interneurons might influence the sensitivity of neuropathic allodynia to GluN2B- versus GluN2A-selective antagonists. To test this, we first examined the responses of SOM^+^ interneurons to the von Frey filament stimulation in the presence or absence of the pharmacological antagonists. We virally expressed the Cre-inducible Ca^2+^ indicator GCaMp6s in spinal SOM^+^ interneurons of male SOM-Cre mice and performed in vivo fiber photometry recordings (Fig 5A). At day 7 post-SNI, the application of innocuous (0.16 g) von Frey filament to the plantar surfaces of the injured paws evoked Ca^2+^ transient increase (Fig 5B). Intraperitoneal application of ifenprodil (10 mg/kg) [43–45] greatly attenuated the Ca^2+^ transients evoked by the von Frey filament stimuli (Fig 5B). By comparison, intraperitoneal injection of TCN-201 (10 mg/kg) [46,47] generated a weaker inhibition of Ca^2+^ transients than ifenprodil (Fig 5B). These data suggested that GluN2B receptors were important for the peripheral mechanosensory inputs to activate spinal SOM^+^ population early after the neuropathy. In female mice, we also observed that ifenprodil inhibited the responses of spinal SOM^+^ population to a greater degree than TCN-201 (Fig 5D). In contrast to these observations early after the neuropathy, TCN-201 produced a more potent inhibition of Ca^2+^ signals than ifenprodil at day 28 post-SNI in male (Fig 5C) and female mice (Fig 5E), possibly due to the subunit switch.

We then compared the effects of intrathecally administered ifenprodil and TCN-201 on mechanical allodynia at day 7 or 28 after SNI. In male mice, ifenprodil elevated the von Frey thresholds (Fig 5F) and alleviated spontaneous pain behaviors (Fig 5H) to a greater extent than TCN-201 at day 7, suggesting the GluN2B hyperfunction in the initiation of neuropathic pain. TCN-201 was, however, more effective than ifenprodil in the pain suppression at day 28 (Fig 5G and 5I). In female mice, ifenprodil generated a more potent action than TCN-201 in elevating the PWT values (Fig 5J) and reducing the spontaneous pain behaviors at day 7 post-SNI (Fig 5L). At day 28 post-SNI, the analgesic effect of ifenprodil in female mice was less prominent than that of TCN-201 (Fig 5K and 5M). These data suggested that the development of neuropathic pain altered the analgesic efficacy of GluN2B versus GluN2A antagonists, a process that was, at least in part, attributable to the nerve injury-induced switch of NMDAR subunit composition at the first-order synapses between the primary afferent fibers and spinal SOM^+^ interneurons.

### RIN1 knockout blocked the GluN2B-to-GluN2A switch during neuropathic pain

**Fig 6:**
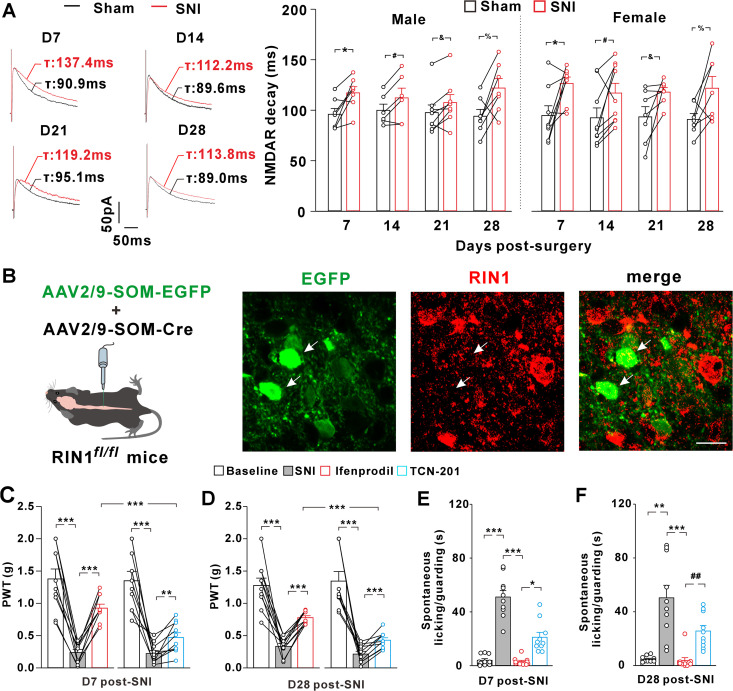
RIN1 knockout blocked the nerve injury from inducing the synaptic GluN2B-to-GluN2A switch in spinal cord SOM^+^ interneurons. **(A)** Comparison of NMDAR-EPSC decay kinetics at days 7-28 after sham and SNI surgery in male (*P = 0.014, ^*#*^*P* = 0.036, ^&^*P* = 0.03, ^%^*P* = 0.026, Paired Student *t* test) and female cKO-RIN1 mice (*P = 0.024, ^*#*^*P* = 0.034, ^&^*P* = 0.048, ^%^*P* = 0.038, paired Student *t* test). n = 6-9 neurons from 2-3 mice per group. **(B)** Targeted deletion of RIN1 by intraspinal injection of AAV2/9-SOM-Cre and AAV2/9-SOM-EGFP in RIN1^*fl/fl*^ mice. Arrows indicated EGFP^+^ neurons with RIN1 deleted. Scale bar, 10 µm. **(C and D)** Ifenprodil was more effective than TCN-201 in elevating PWTs of RIN1-deficient mice at day 7 (C) and day 28 (D) after SNI. **P = 0.003, ***P < 0.001 (repeated measurement with post hoc Bonferroni test). *n* = 10 male and female mice per group. **(E and F)** Ifenprodil was more effective than TCN-201 in alleviating spontaneous pain behaviors of RIN1-deficient mice at day 7 (E) and day 28 (F) after SNI surgery. *P = 0.018, **P = 0.002, ^*##*^*P* = 0.001, ***P < 0.001 (Kruskal–Wallis test). n = 10 male and female mice per group. The data underlying this figure can be found in S1 Data.

To test whether RIN1 was required for the nerve injury-induced subunit switch, we recorded NMDAR synaptic currents on SOM^+^ interneurons at different time points after SNI surgery on the left sciatic nerves of cKO-RIN1 mice. Sham surgery was performed on the right nerves. In male and female mice, SNI prolonged the decay kinetics of NMDAR-EPSCs on day 7 when compared to sham surgery (Fig 6A). However, the RIN1 knockout mice failed to exhibit the time-dependent speeding of NMDAR-EPSC decay (Fig 6A). At days 14, 21, and 28 after SNI, the decay kinetics of NMDAR-EPSCs remained significantly slower when compared to sham controls (Fig 6A), suggesting that RIN1 deletion impeded the switch of NMDAR subunit composition.

We then examined the effect of RIN1 knockout on pain behaviors. To ablate RIN1 selectively on spinal SOM^+^ interneurons, we infused the AAV vectors that drove the expression of Cre recombinase and EGFP under the control of somatostatin promoter (AAV2/9-SOM-Cre and AAV2/9-SOM-EGFP) into spinal cord of RIN1^*fl/fl*^ mice (Fig 6B). The knockdown of RIN1 was verified by the absence of RIN1 immunoreactivity in SOM^+^ neurons (Fig 6B). At both days 7 and 28 after SNI, intrathecal ifenprodil treatment potently inhibited the neuropathic allodynia (Fig 6C and 6D) and spontaneous pain behaviors (Fig 6E and 6F). Compared to ifenprodil, TCN-201 was less effective in the pain suppression at each time point (Fig 6C–6F), suggesting that the impeded switch following RIN1 deletion reduced the analgesic efficacy of GluN2A inhibition, especially at the later stage of neuropathic pain. Taken together, our data revealed a critical role of RIN1 in the activity-dependent switch of synaptic NMDAR subunit composition during both synapse development and neuropathic pain.

## Discussion

Previous studies have investigated the spatiotemporal distribution of RIN1 in the brain, and found the highest expression in the mature forebrain regions, including the hippocampus, amygdala, and cortex [35,36,38,48]. Here we found a developmental increase of RIN1 expression in spinal cord SOM^+^ interneurons. RIN1 accumulated at PSD-95^+^ postsynaptic density, suggesting a possible role in the modification of postsynaptic function. Electrophysiological recordings performed ex vivo in spinal cord slices from conditional RIN1 knockout mice showed intact synaptic responses mediated by either AMPARs or NMDARs, supporting that RIN1 didn’t determine the synaptic number of ionotropic glutamate receptors in live tissues [35].

The GluN2B-to-GluN2A switch is critical for synaptogenesis and development, and has been well-characterized in a line of brain regions [1,16,18,20]. At mature synapses, the GluN2A receptors dominate the NMDAR synaptic responses, whereas the GluN2B receptors are primarily localized at extrasynaptic sites [49]. Recent studies have performed the electrophysiological recordings of mEPSCs on spinal cord neurons, possibly including both excitatory and inhibitory interneurons, and implicate that the molecular identity responsible for NMDAR synaptic responses does not change at lamina II synapses across early postnatal development [25], with GluN2B receptors conserved at spinal synapses throughout neuronal maturation [13,25]. The GluN2D-containing NMDARs, expressed in neuron subtypes such as GABAergic interneurons [50], also persist at mature glutamatergic synapses [13,25]. The current study specifically examined the primary afferent synapses on lamina II SOM^+^ interneurons and found a developmental decline of ifenprodil-sensitive GluN2B component of NMDAR synaptic responses and a coincident increase of TCN-201-sensitive GluN2A component in both male and female mice. This result was consistent with previous recordings on lamina I projection neurons, identifying GluN2A receptors as the major contributor to monosynaptic NMDAR currents at primary afferent synapses [11]. Our data suggested that, similar to the observations in the brain regions such as the hippocampus, the developmental switch of NMDAR subunit composition also occurred at spinal cord synapses, at least at primary afferent-innervated synapses onto SOM^+^ interneurons. Of importance was that genetic knockout of RIN1 from SOM^+^ interneurons blunted the replacement of GluN2B by GluN2A receptors during development, suggesting an important role of postsynaptic RIN1 in driving the GluN2B-to-GluN2A switch.

The subunit switch has been proposed to depend on several factors, including the expression level of GluN2A, discrete biological properties of GluN2 C-terminal domains, and specific signaling cascades initiated by postsynaptic receptors, including the metabotropic glutamate receptor-5 and ionotropic NMDARs [4,12,16,17,22]. Our data showed that RIN1 interacted directly with GluN2A, but not GluN2B subunit. The SH2 domain-containing N-terminal region was sufficient to interact with GluN2A and was responsible for the incorporation of GluN2A receptors onto plasma membrane. The GluN2A-associated RIN1 might act through its Rab5 GEF activity to internalize the pre-existing GluN2B receptors from the cell surface, ultimately manifesting as the developmental switch from GluN2B- to GluN2A receptors at synapses. In the absence of Rab5 GEF activity, RIN1 preserved the ability to insert GluN2A receptors onto plasma membrane but lost the ability to induce the endocytosis of GluN2B receptors.

In spite of the fact that the developmental switch of NMDAR subunit composition reduces synaptic GluN2B abundance at mature synapses, considerable evidence has indicated the importance of GluN2B receptors in a series of neuropsychiatric disorders in adulthood [3,12,51,52]. In the context of chronic pain, the nerve or tissue injury rapidly recruits GluN2B receptors back to the mature synapses of spinal cord nociceptive neurons, potentiating GluN2B-mediated neurotransmission and triggering GluN2B-dependent pain sensitization [3–5]. Pharmacological inhibition of spinal GluN2B receptors alleviates the pain symptoms early after lesions [6,7]. Several post-translational modifications, including the phosphorylation and ubiquitylation, have been implicated in the GluN2B accumulation at synapses during pathological pain [4,7]. Our data illustrated that the nerve injury caused a rapid retention of GluN2B receptors at primary afferent synapses onto SOM^+^ interneurons in both wild-type and RIN1 knockout mice, implying that RIN1 was dispensable for the synaptic trapping process of GluN2B receptors early after the nerve injury. With the development of neuropathic pain, we detected a significant increase of RIN1 protein in spinal SOM^+^ interneurons, which initiated a new round of NMDAR subunit switch at mature synapses between primary afferents and SOM^+^ interneurons. The nerve injury-induced NMDAR subunit switch exhibited no sex difference. As a result, the sensitivity of NMDAR synaptic responses to ifenprodil was decreased later after the nerve injury in both male and female mice, a phenomenon that was not observed in RIN1 knockout mice. Given the lower mobility of GluN2A than GluN2B receptors at postsynaptic membranes [53–55], the nerve injury-induced switch might underpin the persistence of chronic pain. The pathological switch of NMDAR subunit composition changed the sensitivity of neuropathic allodynia to the antagonists targeting different NMDAR subunits. In comparison with the GluN2B inhibition exhibiting potent analgesic action at day 7 after nerve injury, the GluN2A inhibition might represent a more effective strategy in the alleviation of late-phase neuropathic pain. Notably, the nerve injury-induced NMDAR subunit switch was synapse-specific, which didn’t occur at primary afferent synapses onto GABAergic interneurons. We found that the RIN1 expression in the inhibitory interneurons underwent no significant change after the nerve injury.

It is worthwhile to mention that we showed the nerve injury-induced NMDAR subunit switch at ipsilateral synapses between primary afferents and SOM^+^ interneurons by using the contralateral synapses as control. Behaviorally, we observed mechanical allodynia on the ipsilateral sides after the SNI surgery, with the nociceptive thresholds on the contralateral sides unaltered. However, ipsilateral nerve injury is reported to induce contralateral hyperalgesia in some contexts [56]. Therefore, the contralateral side was not ideal to compare the synaptic alteration after peripheral nerve injury. Additional studies are warranted to confirm the nerve injury-induced NMDAR subunit switch at primary afferent synapses onto SOM^+^ interneurons by using sham cohorts as controls.

Taken together, our data revealed an important role of RIN1 in driving GluN2B-to-GluN2A switch during the synaptic maturation and the development of neuropathic pain in spinal cord SOM^+^ interneurons. We provided a new insight into the molecular mechanisms that determined the synaptic NMDAR subunit stoichiometry. These results might have important implications for the neuropsychiatric diseases associated with the dysregulation of synaptic GluN2 subunits.

## Methods and materials

### Ethics statement

The animal experiments in this study were conducted in accordance with the guidelines of the Animal Care and Use Committee of Lanzhou University (No. EAF2024018) and the National Institutes of Health guide for the care and use of Laboratory animals (NIH Publications No. 8023).

### Animals

The adult male and female C57BL/6J mice (8−10 weeks old) were obtained from the Experimental Animal Center of Lanzhou University (NO. SCXK(gan) 2023−0003). The RIN1^*fl/fl*^ mice were purchased from GemPharmatech (#GPS00001659; Suzhou, China). The SST^*tm2.1(cre)Zjh*^/J (SOM-Cre) mice (JAX #013044), B6.Cg-*Gt(ROSA)26Sor*^*tm14(CAG-tdTomato)Hze*^/J (Ai14) mice (JAX #007914) and B6J.129S6(FVB)-*Slc32a1*^*tm2(cre)Lowl*^/MwarJ (Vgat-Cre) mice (JAX# 028862) were purchased from the Jackson laboratory. The SOM-Cre and Ai14 reporter mice were crossed to label SOM^+^ neurons with tdTomato. RIN1 was knocked out by crossing RIN1^*fl/fl*^ with SOM-Cre mice. To delete RIN1 in tdTomato-labeled SOM^+^ neurons, the RIN1^*fl/fl*^ mice were crossed to SOM-Cre and Ai14 mice. The animals were housed three to four per cage on a 12 h light/dark cycle with free access to food and water.

### Virus, expression constructs, and reagents

AAV2/9-EF1α-DIO-EGFP (6 × 10^12^ vg/ml), AAV2/9-EF1α-DIO-RIN1(WT)-EGFP (8 × 10^12^ vg/ml), AAV2/9-EF1α-DIO-RIN1(1–221)-EGFP (1.8 × 10^13^ vg/ml), and AAV2/9-EF1α-DIO-RIN1(E574A)-EGFP (8 × 10^12^ vg/ml) were purchased from Sunbio Biomedical Technology (Shanghai, China). AAV2/9-EF1α-DIO-GCaMp6s (5.4 × 10^12^ vg/ml) was purchased from BrainVTA (Wuhan, China). The RIN1(1–221) construct encoded the amino acids 1 to 221 of mouse RIN1. The site-directed mutagenesis was used to generate RIN1(E574A) mutant in which the glutamic acid at residue 574 was substituted with alanine. The AAV2/9-SOM-EGFP (2 × 10^12^ vg/ml) and AAV2/9-SOM-Cre (5 × 10^12^ vg/ml) were purchased from BrainVTA (Wuhan, China). The recombinant adenovirus (10^10^ plaque-forming units/ml) encoding RIN1 and/or EGFP was purchased from Yingrun Biotechnologies (Changsha, China). Ifenprodil, picrotoxin, 6-Cyano-7-nitroquinoxaline-2,3-dione (CNQX) and N-[[4-(benzamidocarbamoyl)phenyl]methyl]-3-chloro-4-fluorobenzenesulfonamide (TCN-201) were purchased from Sigma-Aldrich. Strychnine and tetrodotoxin (TTX) were purchased from Absin (Shanghai, China).

### Viral injection and drug delivery

The mice were deeply anesthetized with sodium pentobarbital (*i.p.*, 90–120 mg/kg) and mounted on a stereotaxic apparatus. The spinal cord was exposed by a laminectomy [32,57]. A glass pipette filled with the viral vector was mounted on a microsyringe pump to deliver the virus (300 nl) at a speed of 50 nl/min. The pipette tip (40 μm in diameter) was positioned at a depth of 0.2 to 0.3 mm from the dorsal surface of L4-L5 lumbar segment and 0.5 mm apart from the midline. Three injections (0.5 mm apart) were conducted on each side. After the injection, the muscle and skin were closed. Intrathecal injection of drug (5 μl) was administered via direct lumbar puncture as described previously [32,57].

### Animal model of neuropathic pain

Spared nerve injury (SNI) was performed as described previously [32,57]. Briefly, the sciatic nerve was exposed under sodium pentobarbital anesthesia. The common peroneal and tibial nerves were ligated with 5.0 silk sutures and transected. Thereafter, a 2–3 mm section distal to the ligation was removed. The sural nerve was left intact during the surgery. For sham mice, the nerves were exposed without any lesion. The muscle and skin were then closed in layers.

### Behavioral experiments

All behavioral tests were performed blindly. To measure mechanical pain, a series of von Frey filaments was used to stimulate the lateral plantar surface of hindpaws, and 50% paw withdrawal threshold was calculated using the up-down method [32]. To assess spontaneous pain behaviors [32,58], the mice were placed in a transparent plexiglas chamber (18 × 18 × 22 cm). After 30 min of habituation, we videotaped the behaviors and analyzed the time spent on licking and guarding hindpaws over a 30-min period with SuperMaze software (XinRun Information Technology, Shanghai, China).

### Fiber photometry recording

A Triple Color Multi-Channel fiber photometry system (QAXK-FPS-SS-LED-CH, Thinker Tech, Nanjing, China) was used to record calcium signals of SOM^+^ subpopulation in vivo. After 14 days of intraspinal viral injection, a hole was drilled through the transverse processes of vertebra bone to implant an optical fiber (diameter: 200 μm; numerical aperture: 0.22; length: 4.0 mm) above the injection site. The optical fiber was affixed with a skull-penetrating screw and dental acrylic. After 7 days of recovery, saline was intraperitoneally injected 1 h before the plantar surfaces were stimulated by the von Frey filament (0.16 g) to obtain the fiber photometry data. After 6 h, ifenprodil (10 mg/kg) or TCN-201 (10 mg/kg) was intraperitoneally injected to observe the changes of the filament-evoked calcium signals of spinal SOM^+^ interneurons [59,60].

### Immunohistochemistry

The mice were anesthetized by sodium pentobarbital (90–120 mg/kg, *i.p.*) and perfused through the ascending aorta with 20 ml of ice-cold saline, followed by 10 ml of ice-cold paraformaldehyde (4%). The spinal cord was dissected out and post-fixed with 4% paraformaldehyde for 2 h at 4°C. After cryoprotection for 12 h in 30% sucrose at 4°C, the transverse sections (40-μm thickness) of L4-L5 lumbar segments were cut on a vibratome (VT1200S, Leica). The free-floating sections were blocked with phosphate-buffered saline (PBS) containing 1% normal goat serum (NGS) and 0.25% Triton X-100 at 4°C overnight. The sections were incubated with primary antibodies for 24–36 h at 4°C. After three washes with PBS, the sections were incubated with Alexa 488- or Cy3-conjugated secondary antibodies (1:500; Invitrogen, Camarillo, CA) for 2 h at room temperature. The images were captured by a confocal laser scanning microscope (STELLARIS 5, Leica). Primary antibodies used in this study included the mouse anti-RIN1 antibody (1:200; #H00009610-B01P, Novus Biologicals, Littleton, CO), rabbit anti-RIN1 antibody (1:200; #bs-6094R, Biosynthesis Biotechnology, Beijing, China), and mouse anti-PSD-95 antibody (1:200; #51-6900, Invitrogen).

For the surface staining of GluN2A and GluN2B, the spinal sections were prepared as above and blocked with PBS containing 1% NGS without Triton X-100 at 4°C overnight. The surface proteins were labeled by a rabbit antibody against the N-terminal region of GluN2B (1:200, #AGC-003, Alomone Labs, Jerusalem, Israel) or GluN2A (1:200, #480031, Invitrogen) for 24–36 h at 4°C. After three washes with PBS, the slices were incubated with Cy3-conjugated goat anti-rabbit secondary antibody for 2 h at room temperature before image capture.

### Proximity ligation assay

The transverse spinal sections (40-μm thickness) were prepared as above. Fluorescence Proximity ligation (PLA) assay was performed using the Duolink in situ fluorescence kit (#DUO92001, #DUO92005, #DUO92008; Sigma-Aldrich) in accordance with the manufacturer’s instructions [32,61,62]. In brief, the spinal slices were incubated for 10 min at room temperature with the antigen retrieval solution (#P0090, Beyotime). The sections were permeabilized with PBS containing 0.25% Triton X-100 for 12 h, blocked for 60 min at 37°C with the Duolink blocking solution, and incubated for 24 h at 4°C with a mixture of mouse anti-RIN1 antibody (1:100; #H00009610-B01P, Novus Biologicals) and rabbit anti-GluN2A (1:100; #07–632; Millipore; Temecula, CA, USA) or rabbit anti-GluN2B antibody (1:100; #AB1557, Millipore). All the primary antibodies were diluted with 1 × Duolink antibody diluent. After washing with 1 × wash buffer A, the slices were incubated with the Duolink In situ PLA probe anti-rabbit PLUS (1:5) and anti-mouse MINUS (1:5) at 37°C for 1 h in a humid incubator. The remaining PLA probes were washed with 1 × wash buffer A. The slices were incubated for 30 min at 37°C with the ligase diluted (1:40) in 1 × ligation buffer in the humid incubator. After washing with 1 × wash buffer A, the polymerase diluted (1:80) with 1 × amplification buffer was incubated with the slices for 100 min at 37°C in the humid incubator. The slices were sequentially washed with 1 × wash buffer B and 0.01 × wash buffer B, and mounted with Duolink In situ mounting medium. The fluorescence images were captured with a confocal laser scanning microscope (STELLARIS 5, Leica).

### Co-immunoprecipitation, GST pull down assay and western blot

The mice were anesthetized by sodium pentobarbital (90−120 mg/kg, *i.p.*). The L4-L5 lumbar spinal cord was dissected into ice-cold, oxygenated (95% O_2_ + 5% CO_2_) artificial cerebrospinal fluid (ACSF; 119.0 mM NaCl, 2.5 mM CaCl_2_, 2.5 mM KCl, 1.3 mM MgCl_2_, 1.0 mM NaH_2_PO_4_, 26.0 mM NaHCO_3_, and 11.0 mM D-glucose, pH 7.4). The spinal sections were lysed in the radioimmunoprecipitation assay (RIPA) buffer [50.0 mM Tris∙HCl (pH 8.0), 150.0 mM NaCl, 1.0 mM EDTA, 1.0% NP-40, 0.1% SDS, 0.5% sodium deoxycholate and phosphatases/proteases inhibitor cocktail (Sigma-Aldrich)]. After centrifugation at 14,000g for 10 min, we collected the supernatant and measured the protein concentration using the Bicinchoninic Acid Assay Kit (#P0012S, Beyotime).

For co-immunoprecipitation, the supernatant was incubated with anti-RIN1 antibody under gentle rotation at 4°C overnight. The Protein A/G-Agarose beads were incubated with the immune complexes for 4 h at 4°C. After extensive washes with RIPA buffer, the immunoprecipitates were resuspended in SDS sample buffer and boiled for 5 min before immunoblotting analysis. For GST pull-down assay [32,63], the GST fusion proteins were expressed in E. coli BL21 cells and affinity purified by glutathione agarose beads (#G0924, Sigma-Aldrich). The supernatant from spinal cord was incubated with the GST proteins bound to the glutathione agarose beads and rotated for 4 h at 4°C. The beads were washed with RIPA buffer and boiled in SDS sample buffer.

The protein samples were subjected to SDS-Polyacrylamide Gel Electrophoresis and transferred to polyvinylidene difluoride membranes. After being blocked with 5% non-fat milk, the membranes were incubated with primary antibodies overnight at 4°C, followed by incubation with horseradish peroxidase-conjugated secondary antibodies (Jackson ImmunoResearch Laboratories, Baltimore, PA). The blots were visualized by the enhanced chemiluminescence (#P0018HS, Beyotime). The primary antibodies used in the current study included the mouse anti-RIN1 antibody (1:200; #H00009610-B01P, Novus Biologicals), mouse anti-GST antibody (#E0019; Anbo Biotechnology, Jiangsu, China), rabbit anti-GluN2B (#AB1557) and anti-GluN2A (#07-632) antibody (Millipore).

### Electrophysiological recordings

The mice were deeply anesthetized with sodium pentobarbital (90–120 mg/kg, *i.p.*). The lumbar segment of spinal cord was dissected into ice-cold oxygenated (95% O_2_ + 5% CO_2_) sucrose solution (50.0 mM sucrose, 95.0 mM NaCl, 7.0 mM MgCl_2_, 1.8 mM KCl, 1.2 mM NaH_2_PO_4_, 0.5 mM CaCl_2_, 26.0 mM NaHCO_3_, 15.0 mM D-glucose, pH 7.4). The transverse slices (300-μm thickness) with or without L4 or L5 dorsal root were cut on a vibratome stage (VT1200S, Leica). The slices were transferred to a recording chamber and perfused (3–5 ml/min) with oxygenated ACSF for at least 0.5 h at 32 °C before recordings. The superficial dorsal horn neurons positive for EGFP or tdTomato were visually identified by using an Olympus BX51WIF microscope fitted with a 40 × water-immersion objective under fluorescence and transmitted light illumination. A glass pipettes (4–8 MΩ) were filled with the internal solution containing 115.0 mM Cesium methanesulfonate, 20.0 mM CsCl, 10.0 mM HEPES, 2.5 mM MgCl_2_, 4.0 mM Na_2_-ATP, 0.4 mM Na-GTP, 0.6 mM EGTA, and 10.0 mM sodium phosphocreatine (pH 7.25, 295–310 mOsm).

To record the AMPAR-EPSCs, the membrane potential was held at −70 mV with an Axon 700B Amplifier, and the attached dorsal root was stimulated by electrical pulses (0.1 Hz) delivered through a suction electrode. To block γ-aminobutyric acid type A (GABA_A_) receptors and glycine receptors, the perfusate was supplemented with picrotoxin (100.0 μM) and strychnine (2.0 μM). The monosynaptic responses were identified based on the constant latency and absence of conduction failure in response to high-frequency electrical stimulation (20 Hz). For mEPSC recordings, TTX (1.0 μM) was also added into the perfusate.

The NMDAR-EPSCs were recorded at +40 mV in the presence of picrotoxin, strychnine, and AMPAR receptor antagonist CNQX (10.0 μM). The peak currents of NMDARs were divided by those of AMPARs to obtain the NMDAR/AMPAR ratios. We analyzed the weighted decay time constant (τW) of NMDAR-EPSCs based on the following formula: τW = (I_f_ × τ_f_ + I_s_ × τ_s_)/(I_f_ + I_s_), in which I_f_ and I_s_ represented the fast and slow components of NMDAR-EPSCs, and τ_f_ and τ_s_ represented the fast and slow decay time constants, respectively. The inhibition of EPSCs produced by ifenprodil or TCN-201 was calculated according to the following formula: inhibition (%) = (pre-drug EPSCs - post-drug EPSCs)/pre-drug EPSCs × 100.

For current-clamp recordings, the glass pipettes had a resistance of 3–5 MΩ when filled with the internal solution (135.0 mM K-gluconate, 3.0 mM KCl, 10.0 mM Hepes, 0.5 mM EGTA, 1.0 mM MgCl_2_, 4.0 mM Mg-ATP, and 0.5 mM Na-GTP, pH 7.2 adjusted with KOH, 290–300 mOsm). Spontaneous action potential firings were recorded at the resting membrane potentials [32]. The signals were filtered at 2 kHz and sampled at 10 kHz.

### Statistical and quantification analysis

All experimental data were expressed as mean ± SEM. The electrophysiological data were analyzed by Clampfit 8.0 or mini-analysis software. The immunofluorescent images were analyzed with Image-Pro Plus 6.0 software. The RIN1 fluorescent intensity was divided by the somatic area to exclude the influence of cell size and normalized to control value. To assay the synaptic localization of RIN1, a dendritic segment in 10-μm length was randomly selected from each EGFP^+^ neuron. The RIN1 puncta overlapping with PSD-95 puncta were counted along the selected segment by using the Analyze Particles tool and the co-localization plug-in tool in Image-Pro Plus 6.0 software. We performed paired Student *t* test or Mann-Whitney U test for two-group comparisons. The Kruskal–Wallis test was used to analyze the data between multiple groups. The data between multiple groups occurring over time were compared by repeated measurement and post hoc Bonferroni test. *P *< 0.05 was considered as the criterion for statistical significance.

## Supporting information

S1 Data Excel spreadsheet containing the numerical data for Figure panels 1B, 2B, 2C, 2D, 2E, 2G, 2H, 2I, 3F, 3H, 3I, 4A, 4B, 4C, 4D, 4E, 4F, 5B-5E, 5F-5M, 6A, 6C-6D, 6E-6F, S1C, S3B-3C, S3E, S4C-4D, and S4E-4F. (XLSX)

S1 Fig Effects of RIN1 ablation on the miniature excitatory postsynaptic currents (mEPSCs) mediated by AMPARs. (A) Immunohistochemistry showed the conditioned knockout of RIN1 in SOM-Cre::RIN1^*fl*/*fl*^::Ai14 (cKO-RIN1) mice compared to SOM-Cre::Ai14 (control) mice. Scale bar, 5 µm. (B) AMPAR-mEPSCs were recorded on spinal cord SOM^+^ interneurons from control and cKO-RIN1 mice at postnatal day 7 (P7), P14, P21, and P28. (C) Comparison of AMPAR-mEPSCs amplitudes (*up*) and frequencies (*down*) recorded in male (*left*) and female mice (*right*). ^ns^*P* > 0.05 (Mann-Whitney U test). *n* = 7–8 neurons from 3–4 mice per group. The data underlying this figure can be found in S1 Data.
(TIF)

S2 Fig Rescuing RIN1 expression in spinal cord SOM^+^ interneurons of SOM-Cre::RIN1^*fl/fl*^ mice. (A) The SOM^+^ neurons, labeled by intraspinal injection of AAV2/9-DIO-EGFP (green), showed the deficiency of RIN1 (red). (B) Intraspinal injection of AAV2/9- DIO-RIN1-EGFP rescued the RIN1 expression. Arrows indicated the EGFP-positive SOM^+^ neurons. Scale bar, 10 µm.
(TIF)

S3 Fig Chemogenetic inhibition of spinal cord SOM^+^ interneurons attenuated neuropathic mechanical allodynia in male and female mice. (A) Schematic of viral injection in the spinal cord of SOM-Cre mice and experimental schedule. (B) Intraperitoneal (*i.p.*) injection of CNO (5 mg/kg) reversed the mechanical allodynia in male mice. The PWT values were measured before (baseline) and after CNO injection on 7–10 days or 25–28 days after SNI (*left*) and sham surgery (*right*). **P = 0.002, ***P < 0.001 (paired Student *t* test). *n* = 6 mice/group. (C) CNO reversed the mechanical allodynia in female mice. **P = 0.001, ***P < 0.001 (paired Student *t* test). n = 6 mice/group. (D) Post hoc immunofluorescent examination verified the mCherry expression in the spinal cord. Scale bar, 100 µm. (E) Bath application of CNO (10 µM) reduced the action potential (AP) firings of SOM^+^ neurons expressing hM4Di. The horizontal bar indicates bath CNO application. The graph showed the changes in AP firings. **P = 0.003 (paired Student *t* test). *n* = 5 cells from 2 mice. The data underlying this figure can be found in S1 Data.
(TIF)

S4 Fig Nerve injury didn’t affect the decay kinetics of NMDARs expressed at primary afferent synapses onto spinal cord GABAergic inhibitory neurons. (A) Schematic of primary afferent inputs to spinal cord SOM^+^ interneurons and GABAergic interneurons driving feedforward inhibition. PN, projection neurons. (B) Scheme for viral injection in the spinal cord of Vgat-Cre mice and experimental schedule. (C and D) Immunohistochemistry for RIN1 in EGFP^+^ GABAergic interneurons at days 7 (D7) and 28 (D28) after sham and SNI surgery in male (C) and female mice (D). n = 20 neurons from 3 mice per group. (E and F) Comparison of the decay kinetics of NMDAR-EPSCs recorded on GABAergic interneurons at days 7 and 28 after sham and SNI surgery in male (E) and female mice (F). n = 8 neurons from 3–4 mice per group. The data underlying this figure can be found in S1 Data.
(TIF)

S1 Raw Images Uncropped western blot gels used for Fig 3A and 3D. (DOCX)

## Abbreviations

AAV: adeno-associated virus
AMPAR: AMPA receptors
DREADD: designer receptor exclusively activated by designer drugs
EGFP: enhanced green fluorescent protein
GEF: guanine nucleotide exchange factor
PBS: phosphate-buffered saline
PLA: proximity ligation assay
RIN1: Ras and Rab interactor 1
SOM+: somatostatin-positive
SNI: spared nerve injury
SH2: Src homology-2
NMDARs: synaptic N-methyl-D-aspartate subtype glutamate receptors
TTX: tetrodotoxin.
