# Supplementary figures and images for: RIN1 regulates developmental and pain-related plasticity in spinal synapses via NMDA receptor subunit trafficking
Source: PLoS Biol. 2025 Dec 2;23(12):e3003516. doi: 10.1371/journal.pbio.3003516 (PMC12671805; doi:10.1371/journal.pbio.3003516)

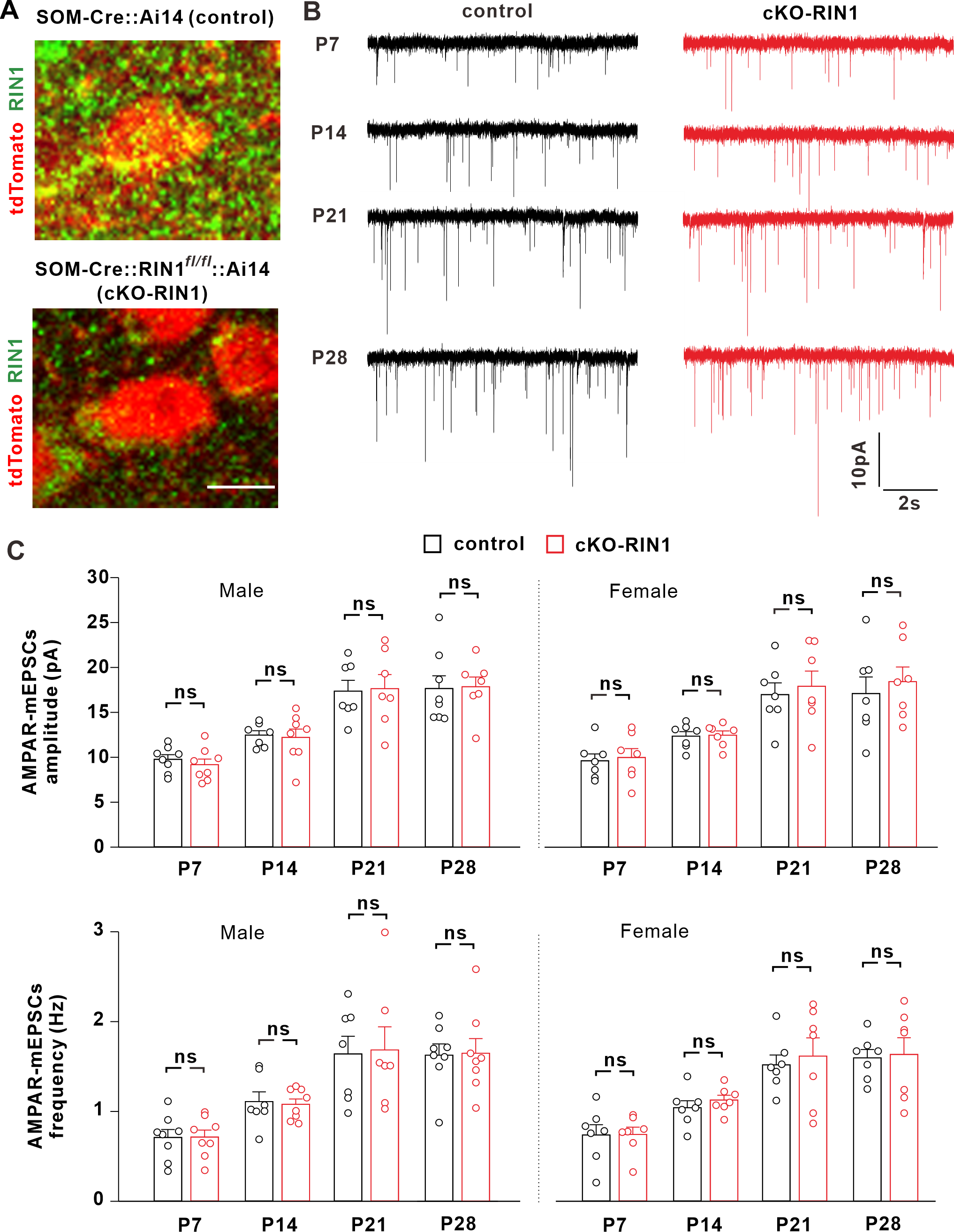

Supplement: S1 Fig — (A) Immunohistochemistry showed the conditioned knockout of RIN1 in SOM-Cre::RIN1fl/fl::Ai14 (cKO-RIN1) mice compared to SOM-Cre::Ai14 (control) mice. Scale bar, 5 µm. (B) AMPAR-mEPSCs were recorded on spinal cord SOM+ interneurons from control and cKO-RIN1 mice at postnatal day 7 (P7), P14, P21, and P28. (C) Comparison of AMPAR-mEPSCs amplitudes (up) and frequencies (down) recorded in male (left) and female mice (right). nsP > 0.05 (Mann-Whitney U test). n = 7–8 neurons from 3–4 mice per group. The data underlying this figure can be found in S1 Data. (TIF) [file pbio.3003516.s002.tif]

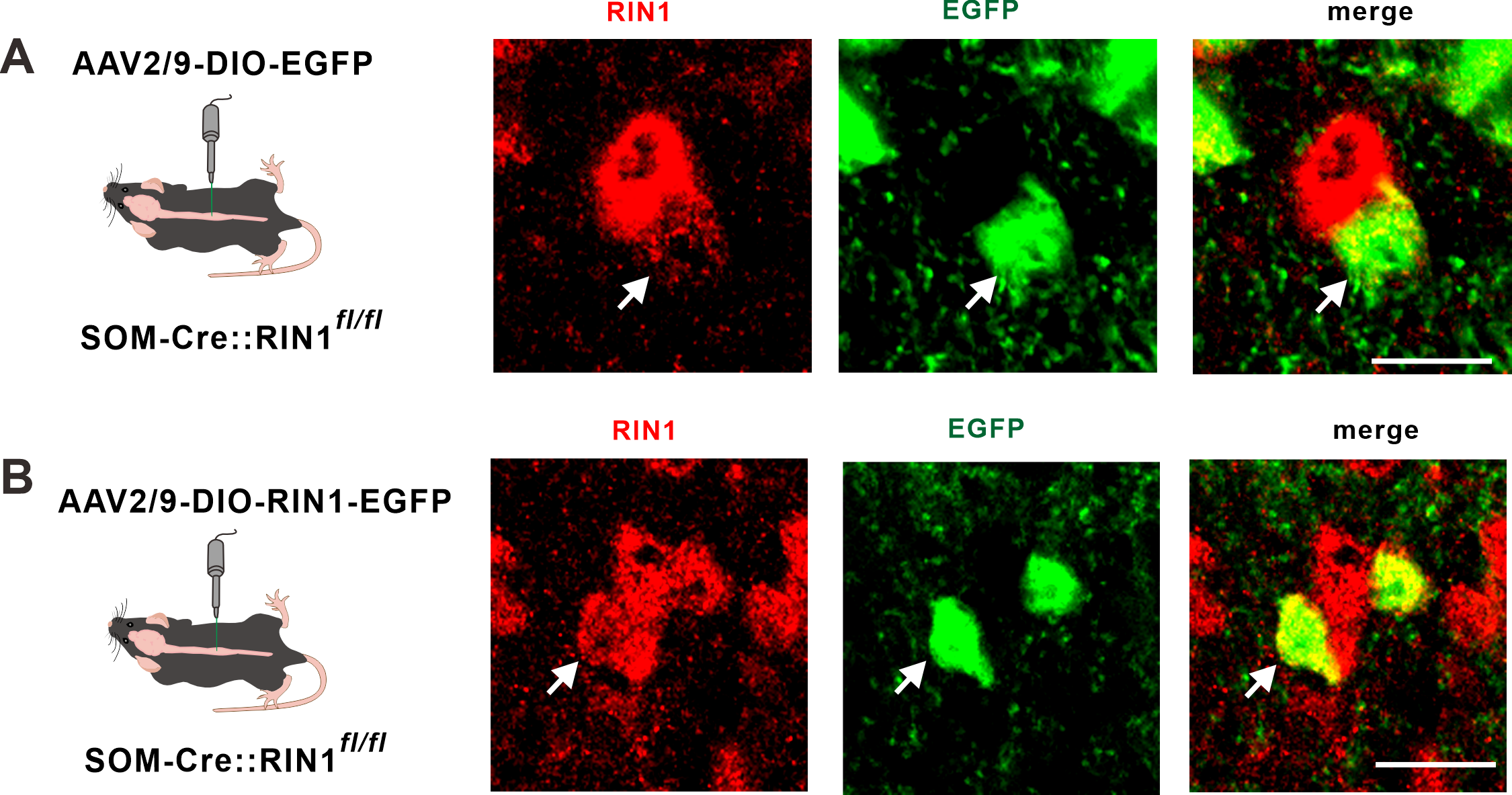

Supplement: S2 Fig — (A) The SOM+ neurons, labeled by intraspinal injection of AAV2/9-DIO-EGFP (green), showed the deficiency of RIN1 (red). (B) Intraspinal injection of AAV2/9- DIO-RIN1-EGFP rescued the RIN1 expression. Arrows indicated the EGFP-positive SOM+ neurons. Scale bar, 10 µm. (TIF) [file pbio.3003516.s003.tif]

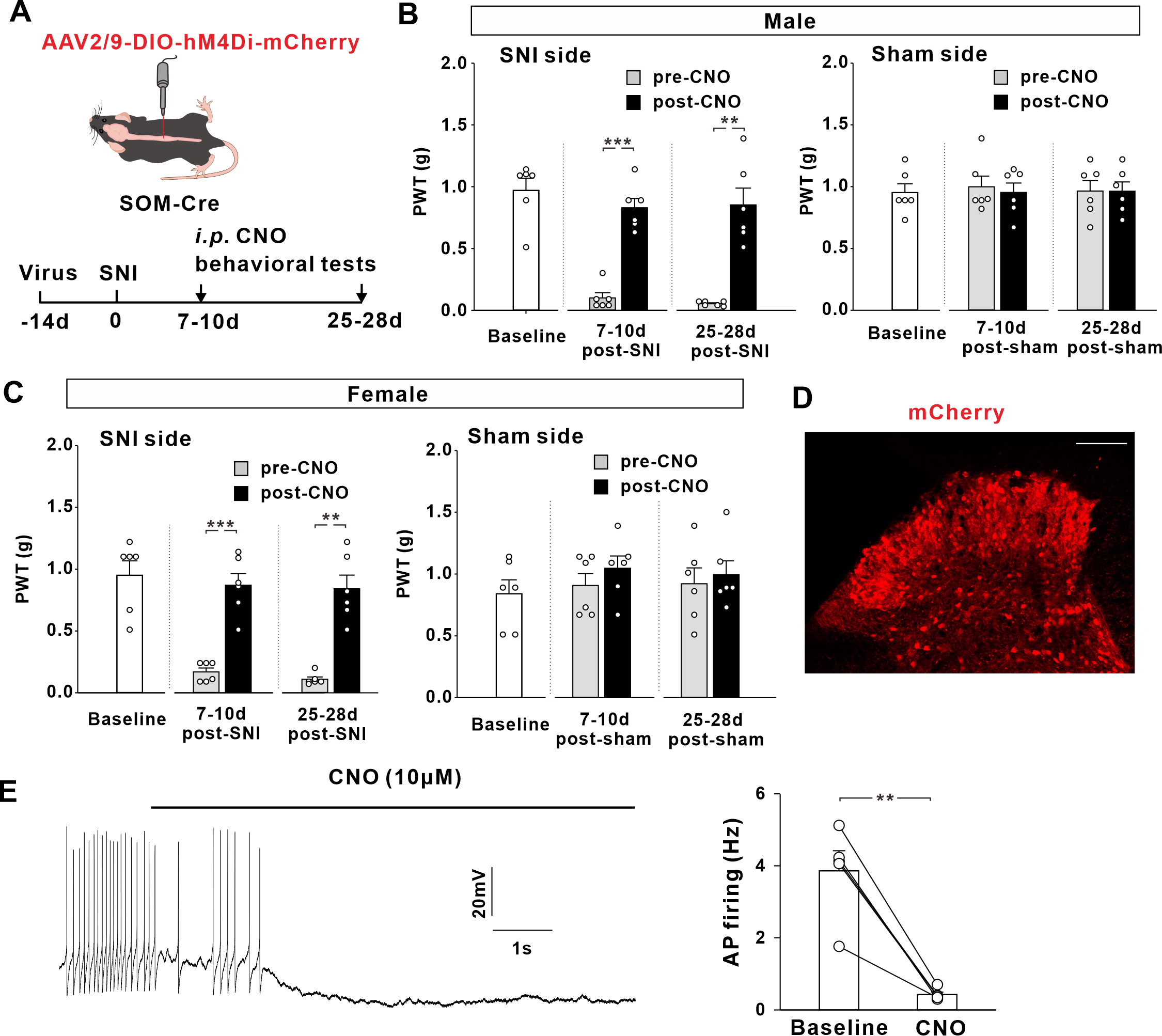

Supplement: S3 Fig — (A) Schematic of viral injection in the spinal cord of SOM-Cre mice and experimental schedule. (B) Intraperitoneal (i.p.) injection of CNO (5 mg/kg) reversed the mechanical allodynia in male mice. The PWT values were measured before (baseline) and after CNO injection on 7–10 days or 25–28 days after SNI (left) and sham surgery (right). **P = 0.002, ***P < 0.001 (paired Student t test). n = 6 mice/group. (C) CNO reversed the mechanical allodynia in female mice. **P = 0.001, ***P < 0.001 (paired Student t test). n = 6 mice/group. (D) Post hoc immunofluorescent examination verified the mCherry expression in the spinal cord. Scale bar, 100 µm. (E) Bath application of CNO (10 µM) reduced the action potential (AP) firings of SOM+ neurons expressing hM4Di. The horizontal bar indicates bath CNO application. The graph showed the changes in AP firings. **P = 0.003 (paired Student t test). n = 5 cells from 2 mice. The data underlying this figure can be found in S1 Data. (TIF) [file pbio.3003516.s004.tif]

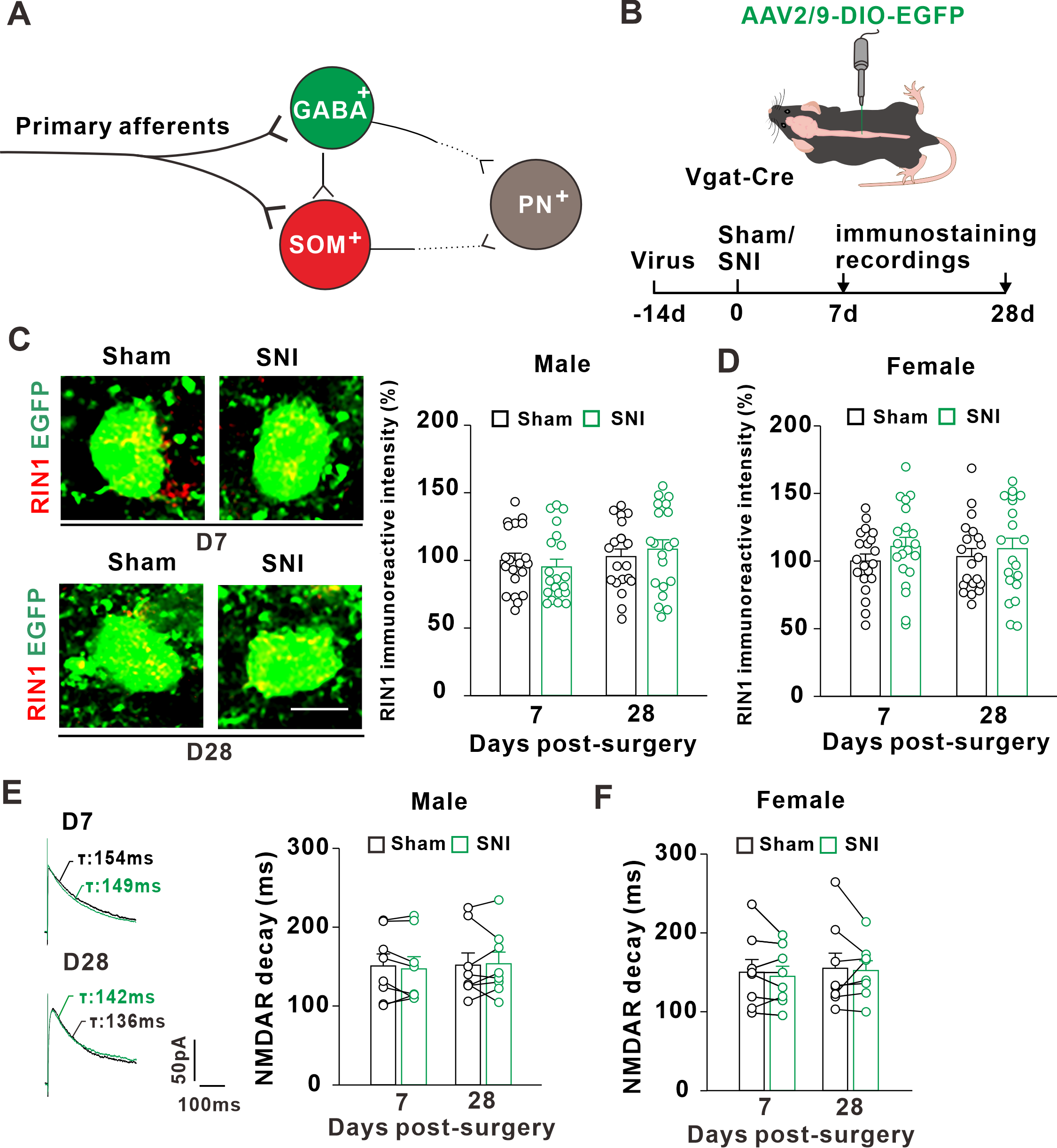

Supplement: S4 Fig — (A) Schematic of primary afferent inputs to spinal cord SOM+ interneurons and GABAergic interneurons driving feedforward inhibition. PN, projection neurons. (B) Scheme for viral injection in the spinal cord of Vgat-Cre mice and experimental schedule. (C and D) Immunohistochemistry for RIN1 in EGFP+ GABAergic interneurons at days 7 (D7) and 28 (D28) after sham and SNI surgery in male (C) and female mice (D). n = 20 neurons from 3 mice per group. (E and F) Comparison of the decay kinetics of NMDAR-EPSCs recorded on GABAergic interneurons at days 7 and 28 after sham and SNI surgery in male (E) and female mice (F). n = 8 neurons from 3–4 mice per group. The data underlying this figure can be found in S1 Data. (TIF) [file pbio.3003516.s005.tif]

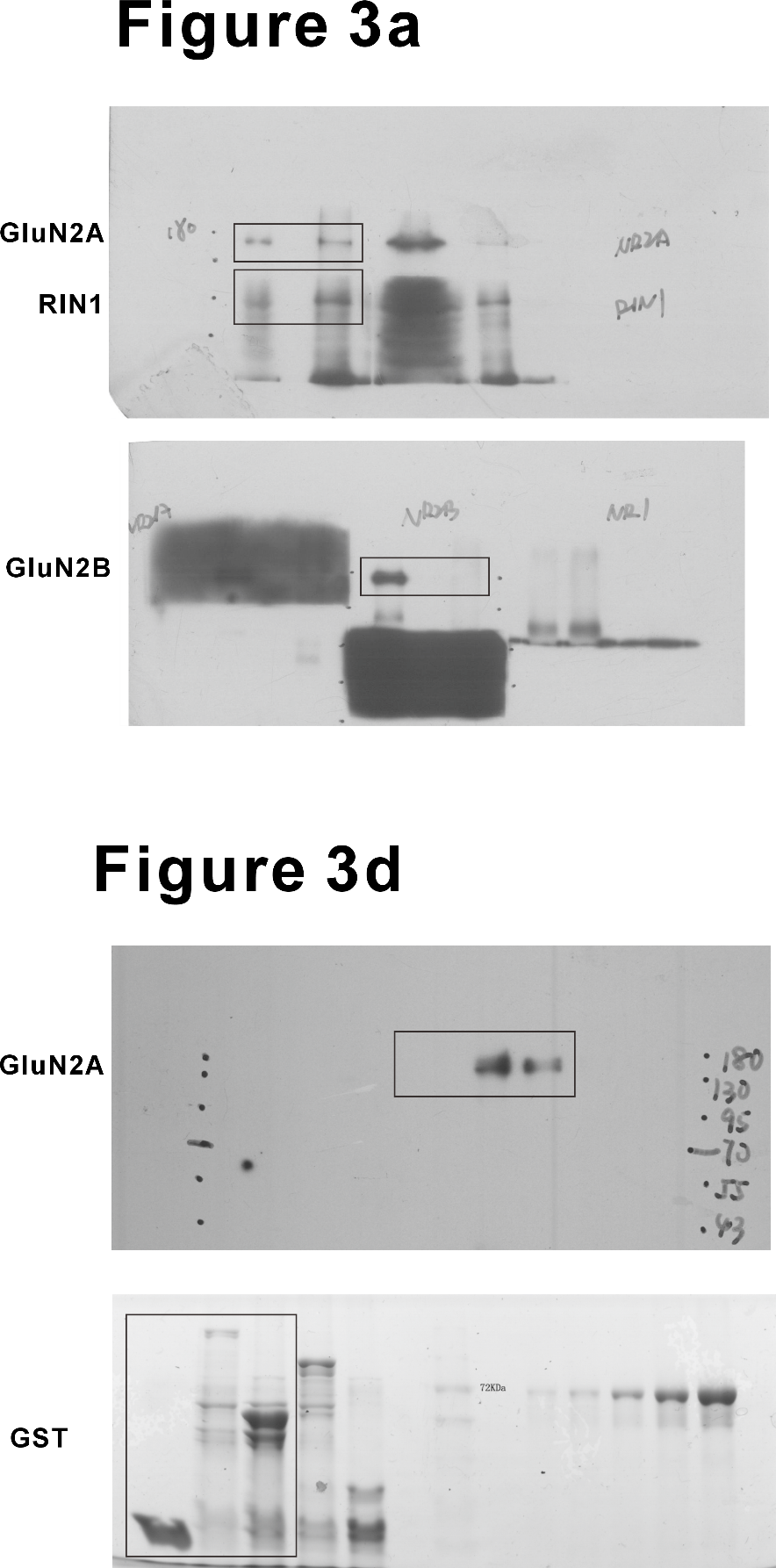

Supplement: S1 Raw Images — (DOCX) [file pbio.3003516.s006.docx]
